# Identification of DARPP-32 as a novel sleep regulator in physiological conditions and experimental Parkinsonism

**DOI:** 10.1016/j.isci.2026.114882

**Published:** 2026-02-02

**Authors:** Clarissa Anna Pisanò, Maria Laura Santino, Alice Russotto, Gilberto Fisone

**Affiliations:** 1 Department of Neuroscience, Karolinska Institutet, 171 77 Stockholm, Sweden

**Keywords:** neuroscience, behavioral neuroscience, Cognitive neuroscience

## Abstract

Sleep disorders are common in Parkinson’s disease (PD) and respond poorly to current pharmacological treatments, partly due to limited understanding of their underlying mechanisms. Using polysomnographic recordings, we investigated the role of dopamine- and cAMP-regulated phosphoprotein 32 kDa (DARPP-32) in sleep-wake regulation and PD-related sleep dysfunction. In naive mice, the selective ablation of DARPP-32 in striatal projection neurons (SPN) co-expressing dopamine D2 and adenosine A2A receptors reduced NREM sleep during the active phase of the circadian cycle, whereas its deletion in dopamine D1 receptor-expressing SPN increased NREM sleep stability during the inactive phase. In a mouse model of PD, excessive daytime sleepiness (EDS), a common non-motor symptom in PD, was abolished by DARPP-32 deletion in D2R/A2AR-expressing SPN but not in D1R-expressing SPN, which also failed to improve sleep fragmentation. Together, these findings identify DARPP-32 as a key regulator of sleep-wake function and a cell-specific target for alleviating PD-related EDS.

## Highlights

• D1R- and A2AR/D2R-expressing neurons regulate NREM sleep via the modulation of DARPP-32
• Loss of DARPP-32 in D1R neurons increases NREM sleep stability
• Loss of DARPP-32 in A2AR/D2R neurons decreases NREM sleep
• DARPP-32 deletion in A2AR/D2R neurons counteracts EDS in experimental PD

## Graphical abstract

**Figure:**
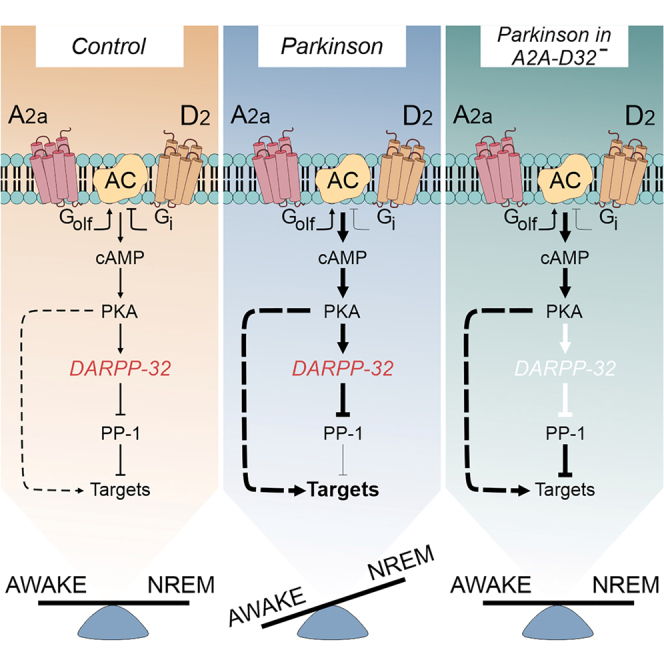

## Introduction

The neurodegenerative processes underlying Parkinson’s disease (PD) are frequently accompanied by sleep disorders, which markedly impair patients’ quality of life. The causal mechanisms linking PD to these comorbidities, affecting both rapid eye movement (REM) and non-REM (NREM) sleep, remain poorly understood, thereby limiting the development of effective therapeutic interventions.^1^^,^^2^^,^^3^

**Figure 1:**
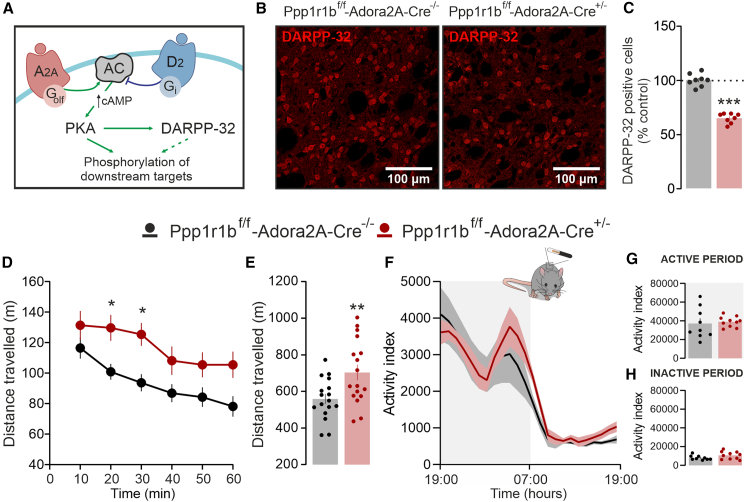
Loss of DARPP-32 in A2AR-expressing neurons increases novelty-induced locomotion (A) Schematic representation of A2AR- and D2R-mediated regulation of DARPP-32, via G_olf_ and G_i_ proteins. (B) Representative confocal images show the loss of DARPP-32 immunoreactive cells in the striatum of a *Ppp1r1b*^f/f^-*Adora2A*-*Cre*^+/−^ mouse in comparison to a *Ppp1r1b*^f/f^-*Adora2A*-*Cre*^−/−^ mouse (scale bars, 100 μm). (C) Quantification of DARPP-32-positive cells expressed as percentage of *Ppp1r1b*^f/f^-*Adora2A*-*Cre*^−/−^ control mice (*n* = 8) and *Ppp1r1b*^f/f^-*Adora2A*-*Cre*^+/−^ (*n* = 8) mice (∗∗∗*p* < 0.001 vs. *Ppp1r1b*^f/f^-*Adora2A*-*Cre*^−/−^; Unpaired *t* test, t = 13.41, df = 14). Data are represented as mean ± SEM. (D and E) Open field test in novel-environment shows (D) time-course of the distance traveled by *Ppp1r1b*^f/f^-*Adora2A*-*Cre*^−/−^ (*n* = 17) and *Ppp1r1b*^f/f^-*Adora2A*-*Cre*^+/−^ (*n* = 17) over 60 min (two-way ANOVA showed a significant effect of time F_5,192_ = 5.788, *p* < 0.001 and genotype F_1,192_ = 31.16, *p* < 0.001, but not time × genotype interaction F_5,192_ = 0.339, *p* = 0.889; ∗*p* > 0.05 ≠ *Ppp1r1b*^f/f^-*Adora2A*-*Cre*^−/−^; Bonferroni post-hoc test), and (E) bar graph of total traveled distance (∗∗*p* = 0.007 vs. *Ppp1r1b*^f/f^-*Adora2A*-*Cre*^−/−^; Unpaired *t* test, t = 2.863, df = 32). Data are represented as mean ± SEM. (F) Locomotion test in familiar environment shows time-course of the distance traveled by *Ppp1r1b*^f/f^-*Adora2A*-*Cre*^−/−^ (*n* = 9) and *Ppp1r1b*^f/f^-*Adora2A*-*Cre*^+/−^ (*n* = 10) over the 24-h circadian cycle recording (two-way ANOVA showed a significant effect of time, F_23,408_ = 17.47 *p* < 0.001, but not genotype F_1,408_ = 3.85, *p* = 0.1754 neither time × genotype interaction F_23,408_ = 1.843, *p* = 0.175; Bonferroni post-hoc test). Data are represented as mean ± SEM. (G and H) Bar graphs show the total distance traveled during the active (G) and the inactive (H) phase. A2A, adenosine receptor type 2A; AC, adenyl cyclase; D2, dopamine receptor type 2; PKA, protein kinase A; cAMP, cyclic adenosine monophosphate; DARPP-32, dopamine- and cAMP-regulated phosphoprotein. Data are represented as mean ± SEM.

In PD, the striatal nucleus of the basal ganglia is profoundly affected by the loss of dopamine innervation caused by the degeneration of the substantia nigra pars compacta (SNc) and, to a lesser extent, the ventral tegmental area (VTA).^4^ Dopamine acts on the two principal populations of striatal projection neurons (SPNs), defined by the expression of dopamine D1 or D2 receptors (D1R and D2R). This segregation enables dopamine to increase the levels of cAMP in D1R-expressing SPN and, conversely, decrease cAMP in those expressing D2R^5^ (cf. Figures 1A and 3A), thereby promoting or reducing the activity of the cAMP-dependent protein kinase (PKA).^5^ Importantly, the inhibitory effect of dopamine on D2R-expressing SPN is exerted by counteracting the enhancement of cAMP-PKA signaling driven by tonically active adenosine A2A receptors (A2ARs) within the same neuronal population.^6^

In addition to their well-established role in various aspects of motor function, SPNs are implicated in the regulation of sleep-wake behavior. In rats, the administration of D1R agonists increases arousal,^7^ whereas D1R antagonism promotes NREM sleep.^8^ Consistent with these findings, recent work in mice shows increased wakefulness following the optogenetic stimulation of D1R-expressing SPN and, conversely, reduced wakefulness following chemogenetic inhibition.^9^

Pharmacological activation of inhibitory postsynaptic D2R in rats promotes wakefulness and reduces NREM sleep.^10^^,^^11^ A similar effect has been shown in mice treated with caffeine, which increases wakefulness via the inhibition of A2AR.^12^ In line with these findings, the chemogenetic activation of A2AR-expressing SPN increases NREM sleep during the active period of the 24-h circadian cycle.^13^ Importantly, previous work indicates that the A2AR-dependent regulation of the sleep-wake cycle is mediated through interaction with D2R-expressing neurons located in the nucleus accumbens, a major component of the ventral striatum.^14^^,^^15^

Despite their important role in the regulation of wakefulness and sleep, relatively little is known about the involvement of SPN in PD-related disturbances, such as excessive daytime sleepiness (EDS) and sleep fragmentation. Pharmacological interventions targeting SPN-associated pathways have produced mixed outcomes. In a mouse model of PD-related sleep abnormalities, the administration of the D2R agonist pramipexole transiently reduced EDS.^16^ In patients with PD, istradefylline, an A2AR antagonist utilized to counteract off-time immobility, has also been reported to reduce EDS.^17^ However, istradefylline therapy is frequently associated with adverse effects, including hallucinations and psychiatric disturbances.^18^ In this context, a deeper understanding of the intracellular mechanisms involved in dopamine- and adenosine-dependent regulation of sleep may facilitate the identification of novel therapeutic targets for PD-related sleep dysfunction.

The regulation of SPN activity by dopamine and adenosine critically depends on dopamine- and cAMP-regulated phosphoprotein of 32 kDa (DARPP-32), a key striatal signaling molecule that enhances protein kinase A (PKA) signaling by inhibiting the dephosphorylation of downstream effector proteins.^19^^,^^20^ DARPP-32 has been implicated in the modulation of locomotor activity and drug addiction downstream of D1R-, D2R-, and A2AR-mediated signaling.^21^^,^^22^^,^^23^^,^^24^^,^^25^ In the present study, we investigated how selective ablation of DARPP-32 in distinct SPN populations affects sleep regulation and assessed the impact of these manipulations on PD-related sleep disturbances in a mouse model.

## Results

### Loss of dopamine- and cAMP-regulated phosphoprotein 32 kDa in A2A receptors-expressing neurons increases novelty-induced locomotion

Mice deficient for DARPP-32 in D2R or A2AR co-expressing SPN were generated using a different driver line (i.e., *Adora2a*-Cre mice) than that employed in previous studies (i.e., *Drd2*-Cre mice; cf.^21^^,^^26^). Accordingly, we first validated their phenotype by assessing striatal DARPP-32 depletion. Immunohistochemical analysis revealed a significant reduction in the number of DARPP-32–positive cells in A2A-D32^-^ mice compared with control, A2A-D32^+^ littermates (Figures 1B and 1C). This finding was confirmed by Western blot analysis showing decreased levels of DARPP-32 in striatal tissue (Figure S1B).

We next examined whether DARPP-32 depletion in A2AR-expressing neurons was associated with enhanced locomotor activity in the open field, as previously reported.^21^ During the first hour of exploration, A2A-D32^-^ mice traveled a significantly greater distance than control mice, indicative of increased novelty-induced locomotion (Figures 1D and 1E).

In parallel experiments, locomotor activity was assessed after habituation. To this end, mice were monitored in their home cages by radio-frequency-based tracking across the entire 24-h cycle.^27^ Under these conditions, habituated A2A-D32^−^ and control A2A-D32^+^ mice exhibited comparable levels of locomotor activity during both the active and inactive phases (Figures 1F–1H).

### Dopamine- and cAMP-regulated phosphoprotein 32 kDa depletion in A2A receptors-expressing neurons reduces NREM sleep during the active phase

**Figure 2:**
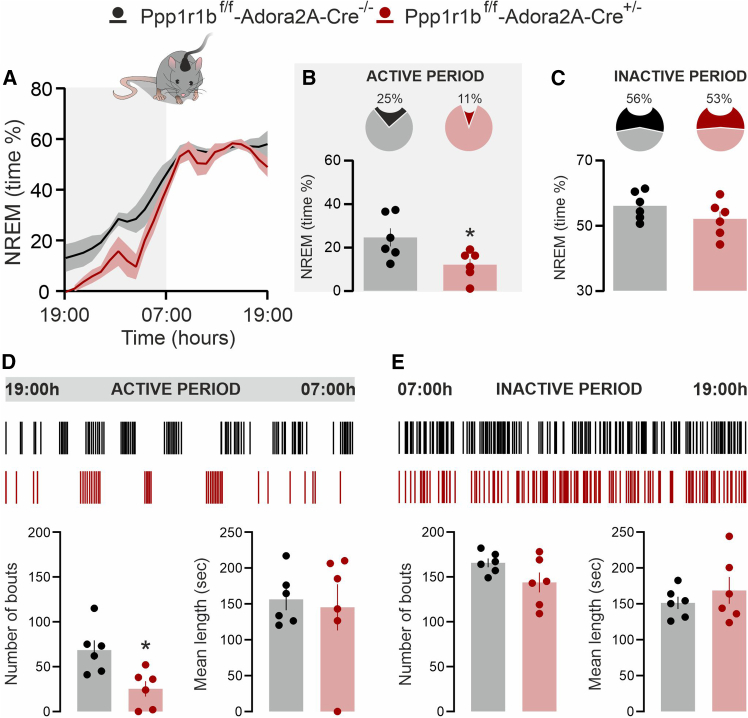
*DARPP-32 depletion in A2A neurons reduces NREM sleep in the active phase* (A) Time-course of NREM sleep in *Ppp1r1b*^f/f^-*Adora2A*-*Cre*^−/−^ and *Ppp1r1b*^f/f^-*Adora2A*-*Cre*^+/−^ mice during the 24-h circadian cycle (graph is expressed as % over total recording time). (B and C) Pie chart (top) and bar graph (bottom) show the average NREM sleep time spent by *Ppp1r1b*^f/f^ (*n* = 6) and *Ppp1r1b*^f/f^-*Adora2A*-*Cre*^+/−^ (*n* = 6) during the active (B) and inactive (C) phase (∗*p* = 0.026 vs. *Ppp1r1b*^f/f^-*Adora2A*-*Cre*^−/−^; Unpaired *t* test, t = 2.617, df = 10). Data are represented as mean ± SEM. (D and E) Upper panels show hypnograms illustrating the distribution of NREM episodes in *Ppp1r1b*^f/f^-*Adora2A*-*Cre*^−/−^ and *Ppp1r1b*^f/f^-*Adora2A*-*Cre*^+/−^ across the active (D) and inactive (E) phases. Bottom panels show the number and length of NREM sleep bouts in *Ppp1r1b*^f/f^-*Adora2A*-*Cre*^−/−^ and *Ppp1r1b*^f/f^-*Adora2A*-*Cre*^+/−^ during the active (D) and inactive (E) phases. ∗*p* = 0.011 vs. *Ppp1r1b*^f/f^-*Adora2A*-*Cre*^−/−^; Unpaired *t* test, t = 3.118, df = 10. Data are represented as mean ± SEM.

Polysomnographic recording of wake and sleep states was performed during the 24-h circadian cycle (Figure 2A). During the active phase (19:00–07:00), A2A-D32^−^ mice exhibited a reduction in NREM sleep compared with control A2A-D32^+^ littermates (Figure 2B). This decrease was attributable to a reduced number of NREM sleep bouts, without a change in mean bout duration (Figure 2D, bottom panels). In contrast, no differences in NREM sleep amount or architecture were detected between A2A-D32^−^ and A2A-D32^+^ mice during the inactive phase (Figures 2C and 2E).

### Loss of dopamine- and cAMP-regulated phosphoprotein 32 kDa in D1 receptor-expressing neurons reduces locomotion

**Figure 3:**
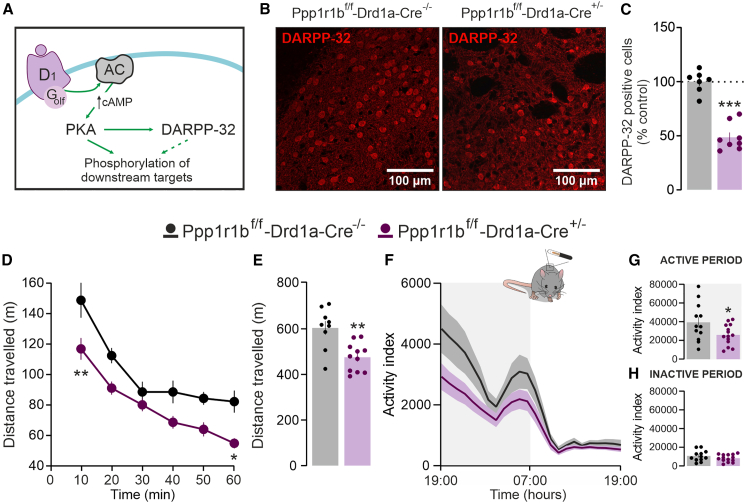
*Loss of DARPP-32 in D1R-expressing neurons reduces locomotion in novel and familiar environments* (A) Schematic representation of the D1R-mediated regulation of DARPP-32, via Golf protein. (B) Representative confocal images show the loss of DARPP-32 immunoreactive cells in the striatum of a *Ppp1r1b*^f/f^-*Drd1a*-*Cre*^+/−^ mouse in comparison to a *Ppp1r1b*^f/f^-*Drd1a*-*Cre*^−/−^ mouse (scale bars, 100 μm). (C) Quantification of DARPP-32-positive cells expressed as the percentage of *Ppp1r1b*^f/f^-*Drd1a*-*Cre*^−/−^ control mice (*n* = 7) and *Ppp1r1b*^f/f^-*Drd1a*-*Cre*^+/−^ mice (*n* = 8) (∗∗∗*p* < 0.001 vs. control; unpaired *t* test, t = 8.650, df = 13). Data are represented as mean ± SEM. (D and E) Open field test in novel-environment shows (D) time-course of the distance traveled by *Ppp1r1b*^f/f^-*Drd1a*-*Cre*^−/−^ (*n* = 9) and *Ppp1r1b*^f/f^-*Drd1a*-*Cre*^+/−^ (*n* = 11) mice over 60 min (two-way ANOVA showed a significant effect of time F_5,108_ = 30.99, *p* < 0.001 and genotype F_1, 108_ = 37.97, *p* < 0.001, but not time × genotype interaction F_5,108_ = 0.85, *p* = 0.514), and (E) bar graph showing total traveled distance (∗∗*p* = 0.001 vs. *Ppp1r1b*^f/f^-*Drd1a*-*Cre*^−/−^; Unpaired *t* test, t = 3.770, df = 18). Data are represented as mean ± SEM. (F) Locomotion test in familiar environment shows time-course of the distance traveled by *Ppp1r1b*^f/f^-*Drd1a*-*Cre*^−/−^ (*n* = 12) and *Ppp1r1b*^f/f^-*Drd1a*-*Cre*^+/−^ (*n* = 14) mice over the 24-h circadian cycle recording (two-way ANOVA showed a significant effect of time F_23,576_ = 17.27, *p* < 0.001 and genotype F_1,576_ = 26.93, *p* < 0.001, but not time × genotype interaction; ∗*p* > 0.05, ∗∗*p* > 0.01 ≠ *Ppp1r1b*^f/f^-*Drd1a*-*Cre*^−/−^; Bonferroni post-hoc test). Data are represented as mean ± SEM. (G and H) Bar graphs showing total distance traveled during the active (G) (∗*p* = 0.042 vs. Control; Unpaired *t* test, t = 2.153, df = 24) and the inactive (H) phase. Abbreviations: D1 dopamine receptor type 1; AC adenyl cyclase; PKA protein kinase A; cAMP cyclic adenosine monophosphate; DARPP-32 dopamine and cAMP-regulated phosphoprotein. Data are represented as mean ± SEM.

In line with previous studies,^21^^,^^26^ immunofluorescence analysis showed a significant reduction of DARPP-32-positive cells in the striatum of D1-D32^-^ mice, compared to D1-D32^+^ control littermates (Figures 3B and 3C). Similar results were obtained by Western blot analysis (Figure S1E).

In contrast to A2A-D32^-^ mice, D1-D32^-^ mice exhibited decreased novelty-induced locomotion in the open field, when compared to their control D1-D32^+^ littermates (Figures 3D and 3E). Following habituation, locomotor activity measured in D1-D32^-^ mice during the active phase was still lower than in control (Figures 3F and 3G). No difference was observed between the two experimental groups during the inactive phase (Figures 3F and 3H).

### Dopamine- and cAMP-regulated phosphoprotein 32 kDa depletion in D1 neurons enhances non-rapid eye movement stability during the inactive phase

**Figure 4:**
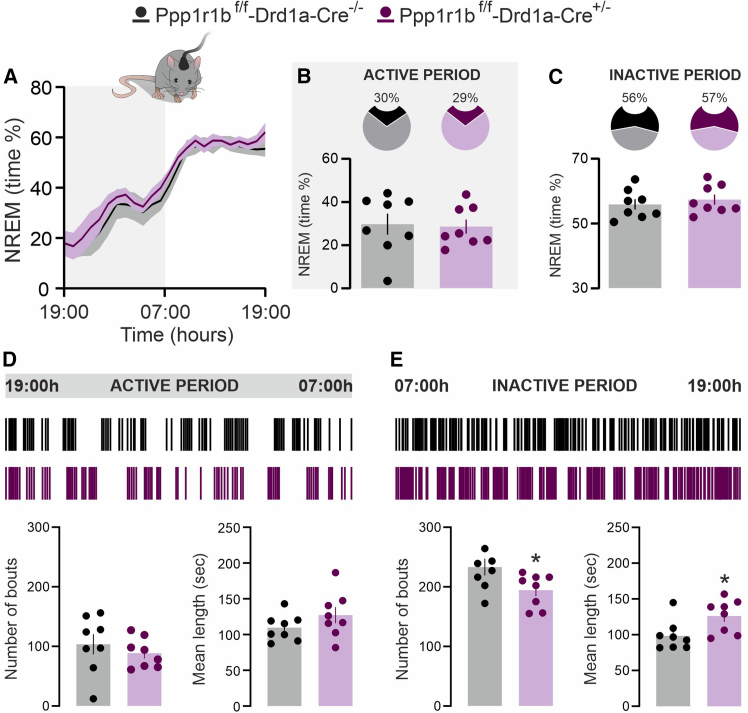
*DARPP-32 depletion in D1 neurons enhances NREM stability during the inactive phase* (A) Time-course of NREM sleep in *Ppp1r1b*^f/f^-*Drd1a*-*Cre*^−/−^ and *Ppp1r1b*^f/f^-*Drd1a*-*Cre*^+/−^ mice, during the 24-h circadian cycle (graph is expressed as % over total recording time). (B and C) Pie chart (top) and bar graph (bottom) show the average NREM sleep time spent by *Ppp1r1b*^f/f^-*Drd1a*-*Cre*^−/−^ (*n* = 8) and *Ppp1r1b*^f/f^-*Drd1a*-*Cre*^+/−^ (*n* = 8) mice during the active (B) and inactive (C) phase. (D and E) Upper panels show hypnograms illustrating the distribution of NREM episodes in *Ppp1r1b*^f/f^-*Drd1a*-*Cre*^−/−^ and *Ppp1r1b*^f/f^-*Drd1a*-*Cre*^+/−^ mice across the active (D) and inactive (E) phases. Bottom panels show the number and length of NREM sleep bouts in *Ppp1r1b*^f/f^-*Drd1a*-*Cre*^−/−^and *Ppp1r1b*^f/f^-*Drd1a*-*Cre*^+/−^ mice during the active (D, ∗*p* = 0.034 vs. *Ppp1r1b*^f/f^-*Drd1a*-*Cre*^−/−^; Unpaired *t* test; t = 2.273, df = 14) and inactive (E, bottom right panel; ∗*p* = 0.025; Unpaired *t* test; t = 2.499, df = 14) phase. Data are represented as mean ± SEM.

Polysomnographic analysis of D1-D32^−^ and D1-D32^+^ mice revealed no differences in total NREM sleep across the 24-h circadian cycle, as assessed by the percentage of time spent in NREM sleep (Figures 4A–4C). However, the analysis of sleep architecture demonstrated that D1-D32^−^ mice exhibited a significant reduction in the number of NREM sleep bouts (Figure 4E, bottom left panel), accompanied by an increase in mean bout duration (Figure 4E, bottom right panel). These alterations, indicative of enhanced NREM sleep stability, were restricted to the inactive phase of the circadian cycle (Figures 4D and 4E).

### Loss of dopamine- and cAMP-regulated phosphoprotein 32 kDa in A2A neurons prevents Parkinson’s disease-related excessive daytime sleepiness

Sleep analyses in naive mice revealed that loss of DARPP-32 in A2AR- or D1R-expressing striatal neurons resulted in distinct alterations of NREM sleep. To determine whether these changes influence PD-related sleep disturbances, we next examined the effects of DARPP-32 ablation in a mouse model of PD that recapitulates EDS and sleep fragmentation.^16^

**Figure 5:**
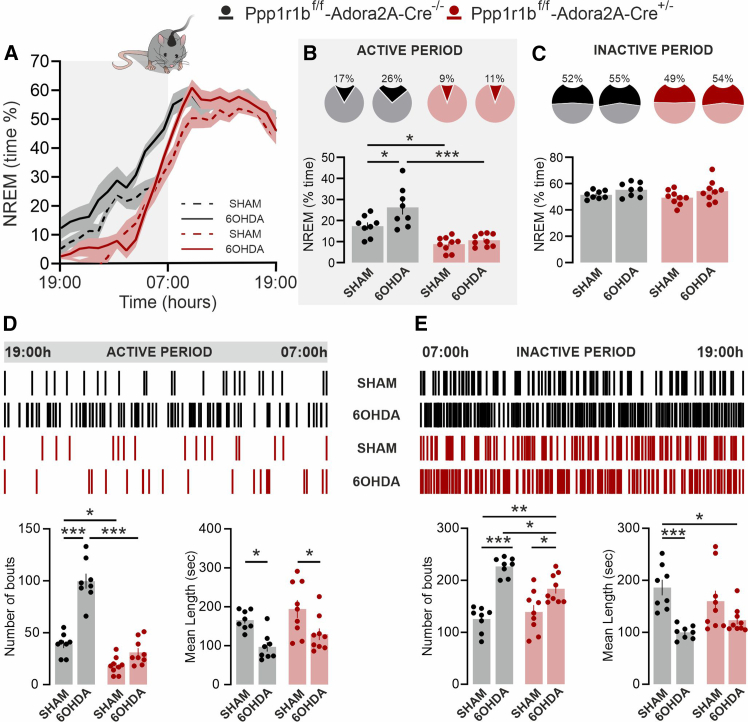
*Depletion of DARPP-32 in A2A neurons prevents EDS* (A) Time course of NREM sleep in PPP1R1B^f/f^ sham-lesion, *Ppp1r1b*^f/f^-*Adora2A*-*Cre*^−/−^ 6-OHDA-lesion, *Ppp1r1b*^f/f^-*Adora2A*-*Cre*^+/−^ sham-lesion, and *Ppp1r1b*^f/f^-*Adora2A*-*Cre*^+/−^ 6-OHDA-lesion mice, during the 24-h circadian cycle (graph is expressed as % over total recording time). (B and C) Pie chart (top) and bar graph (bottom) show the average NREM sleep time spent by *Ppp1r1b*^f/f^-*Adora2A*-*Cre*^−/−^ sham-lesion (*n* = 8), *Ppp1r1b*^f/f^-*Adora2A*-*Cre*^−/−^ 6-OHDA-lesion (*n* = 8), *Ppp1r1b*^f/f^-*Adora2A*-*Cre*^+/−^ sham-lesion (*n* = 9) and *Ppp1r1b*^f/f^-*Adora2A*-*Cre*^+/−^ 6-OHDA-lesion (*n* = 9) mice during the active (B, the two-way ANOVA showed a significant effect of the treatment F (1, 30) = 7.45; ∗*p* = 0.010 and the genotype F (1, 30) = 37.97; ∗∗∗*p* < 0.001, but no interaction; Multiple comparison: *Ppp1r1b*^f/f^-*Adora2A*-*Cre*^−/−^ sham-lesion vs. *Ppp1r1b*^f/f^-*Adora2A*-*Cre*^+/−^ sham-lesion ∗*p* = 0.026; PPP1R1B^f/f^ sham-lesion vs. *Ppp1r1b*^f/f^-*Adora2A*-*Cre*^−/−^ 6-OHDA-lesion ∗*p* = 0.024; *Ppp1r1b*^f/f^-*Adora2A*-*Cre*^−/−^ 6-OHDA vs. *Ppp1r1b*^f/f^-*Adora2A*-*Cre*^+/−^ 6-OHDA-lesion ∗∗∗*p* < 0.001) and inactive (C, the two-way ANOVA showed only a significant effect of the treatment F (1, 30) = 4.857; ∗*p* = 0.035; genotype ns, but no interaction) phase. Data are represented as mean ± SEM. (D and E) Upper panels show hypnograms illustrating the distribution of NREM episodes in *Ppp1r1b*^f/f^-*Adora2A*-*Cre*^−/−^ sham-lesion, *Ppp1r1b*^f/f^-*Adora2A*-*Cre*^−/−^ 6-OHDA-lesion, *Ppp1r1b*^f/f^-*Adora2A*-*Cre*^+/−^ sham-lesion, and *Ppp1r1b*^f/f^-*Adora2A*-*Cre*^+/−^ 6-OHDA-lesion mice across the active (D) and inactive (E) phases. Bottom panels show the number and length of NREM sleep bouts in *Ppp1r1b*^f/f^ sham-lesion, *Ppp1r1b*^f/f^-*Adora2A*-*Cre*^−/−^ 6-OHDA-lesion, *Ppp1r1b*^f/f^-*Adora2A*-*Cre*^+/−^ sham-lesion and *Ppp1r1b*^f/f^-*Adora2A*-*Cre*^+/−^ 6-OHDA-lesion mice during the active (D, *number of bouts*: the two-way ANOVA showed a significant effect of treatment F (1, 30) = 63.41; ∗∗∗*p* < 0.001, genotype F (1, 30) = 92.97; ∗∗∗*p* < 0.001 and interaction F (1, 30) = 26.78; ∗∗∗*p* < 0.001; Multiple comparison: PPP1R1B^f/f^ sham-lesion vs. *Ppp1r1b*^f/f^-*Adora2A*-*Cre*^+/−^ sham-lesion ∗*p* = 0.022; PPP1R1B^f/f^ sham-lesion vs. *Ppp1r1b*^f/f^-*Adora2A*-*Cre*^−/−^ 6-OHDA-lesion ∗∗∗*p* < 0.001; *Ppp1r1b*^f/f^ 6-OHDA vs. *Ppp1r1b*^f/f^-*Adora2A*-*Cre*^+/−^ 6-OHDA-lesion ∗∗∗*p* < 0.001; *mean length*: the two-way ANOVA showed only a significant effect of treatment F (1, 30) = 16.97; ∗∗∗*p* < 0.001, genotype ns and interaction ns; Multiple comparison: *Ppp1r1b*^f/f^-*Adora2A*-*Cre*^−/−^ sham-lesion vs. *Ppp1r1b*^f/f^-*Adora2A*-*Cre*^−/−^ 6-OHDA-lesion ∗*p* = 0.040; *Ppp1r1b*^f/f^-*Adora2A*-*Cre*^+/−^ sham-lesion vs. *Ppp1r1b*^f/f^-*Adora2A*-*Cre*^+/−^ 6-OHDA-lesion ∗*p* = 0.040) and inactive (E, *number of bouts*: the two-way ANOVA showed a significant effect of treatment F (1, 30) = 54.82; ∗∗∗*p* < 0.001, genotype ns and interaction F (1, 30) = 8.26; ∗∗*p* = 0.007; Multiple comparison: *Ppp1r1b*^f/f^-*Adora2A*-*Cre*^−/−^ sham-lesion vs. *Ppp1r1b*^f/f^ 6-OHDA-lesion ∗∗∗*p* < 0.001; *Ppp1r1b*^f/f^-*Adora2A*-*Cre*^−/−^ sham-lesion vs. *Ppp1r1b*^f/f^-*Adora2A*-*Cre*^+/−^ 6-OHDA-lesion ∗∗*p* = 0.001; *Ppp1r1b*^f/f^-*Adora2A*-*Cre*^−/−^ 6-OHDA-lesion vs. *Ppp1r1b*^f/f^-*Adora2A*-*Cre*^+/−^ 6-OHDA-lesion ∗*p* = 0.026; *Ppp1r1b*^f/f^-*Adora2A*-*Cre*^+/−^ 6-OHDA-sham vs. *Ppp1r1b*^f/f^-*Adora2A*-*Cre*^+/−^ 6-OHDA-lesion ∗*p* = 0.015; *mean length*: the two-way ANOVA showed only a significant effect of treatment F (1, 30) = 20.21; ∗∗∗*p* < 0.001, genotype ns and interaction ns; Multiple comparison: *Ppp1r1b*^f/f^-*Adora2A*-*Cre*^−/−^ sham-lesion vs. *Ppp1r1b*^f/f^-*Adora2A*-*Cre*^−/−^ 6-OHDA-lesion ∗∗∗*p* < 0.001; *Ppp1r1b*^f/f^-*Adora2A*-*Cre*^−/−^ sham-lesion vs. *Ppp1r1b*^f/f^-*Adora2A*-*Cre*^+/−^ 6-OHDA-lesion ∗*p* = 0.017) phase. Bonferroni post-hoc test. Data are represented as mean ± SEM.

A2A-D32^+^ mice and DARPP-32-deficient A2A-D32^-^ mice were subjected to either sham- or 6-OHDA-lesion and subsequently examined by 24-h polysomnographic recording. During the active phase, 6-OHDA-lesion A2A-D32^+^ mice exhibited a significant increase in time spent in NREM sleep compared with sham-lesion A2A-D32^+^ controls (Figures 5A and 5B). Further analysis also revealed that, following lesion with 6-OHDA, A2A-D32^+^ mice displayed a marked increase in the number of NREM sleep bouts, accompanied by a decrease in the mean length (Figures 5D and 5E). Altogether, these findings are in line with previous work in naive mice, showing EDS and sleep fragmentation in response to lesion with 6-OHDA.^16^

In contrast to A2A-D32^+^ mice, 6-OHDA lesion in A2A-D32^-^ mice failed to increase NREM sleep compared with either sham-lesion A2A-D32^+^ controls (Figures 5A–5C) or sham-lesion A2A-D32^-^ mice (Figures 5E–5C). The protective effect exerted by DARPP-32 depletion against EDS was mediated by the normalization of the number of NREM sleep bouts (Figure 5D, bottom panels).

During the inactive phase, 6-OHDA-lesion increased the number of NREM sleep bouts in both A2A-D32^+^ and A2A-D32^-^ mice compared with sham-lesion A2A-D32^+^ controls (Figure 5E, bottom left panel). This increase occurred in concomitance with decreased length of NREM sleep episodes (Figure 5E, bottom right panel), resulting in an overall amount of NREM sleep comparable to that of sham-lesioned A2A-D32^+^ mice (Figures 5A and 5C). Collectively, these findings indicate that, in the mouse model of Parkinson’s disease, loss of DARPP-32 in A2AR-expressing neurons mitigates excessive daytime sleepiness without preventing the fragmentation of NREM sleep during the inactive phase.

### Loss of dopamine- and cAMP-regulated phosphoprotein 32 kDa in D1 neurons fails to improve Parkinson’s disease-related sleep disruptions

**Figure 6:**
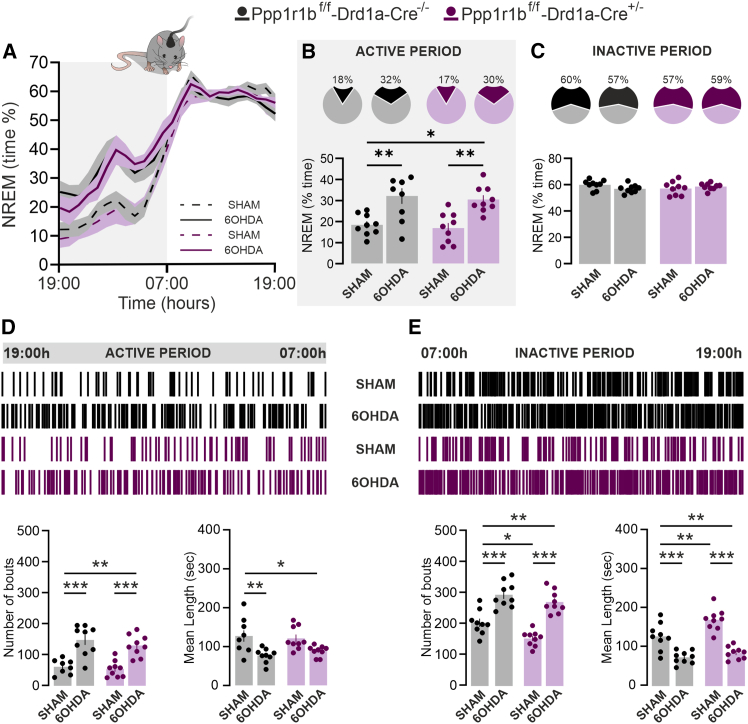
*Depletion of DARPP-32 in D1 neurons does not affect PD-related sleep disruptions* (A) Time course of NREM sleep in *Ppp1r1b*^f/f^-*Drd1a*-*Cre*^−/−^ sham-lesion, *Ppp1r1b*^f/f^-*Drd1a*-*Cre*^−/−^ 6-OHDA-lesion, *Ppp1r1b*^f/f^-*Drd1a*-*Cre*^+/−^ mice sham-lesion, and *Ppp1r1b*^f/f^-*Drd1a*-*Cre*^+/−^ mice 6-OHDA-lesion mice, during the 24-h circadian cycle (graph is expressed as % over total recording time). (B and C) Pie chart (top) and bar graph (bottom) show the average NREM sleep time spent by *Ppp1r1b*^f/f^-*Drd1a*-*Cre*^−/−^ sham-lesion (*n* = 9), *Ppp1r1b*^f/f^-*Drd1a*-*Cre*^−/−^ 6-OHDA-lesion (*n* = 9), *Ppp1r1b*^f/f^-*Drd1a*-*Cre*^+/−^ mice sham-lesion (*n* = 9) and *Ppp1r1b*^f/f^-*Drd1a*-*Cre*^+/−^ mice 6-OHDA-lesion (*n* = 9) mice during the active phase (B; the two-way ANOVA showed a significant effect of treatment F (1, 32) = 27.87; ∗∗∗*p* < 0.001, genotype ns, interaction ns; Multiple comparison: *Ppp1r1b*^f/f^-*Drd1a*-*Cre*^−/−^ sham-lesion vs. *Ppp1r1b*^f/f^-*Drd1a*-*Cre*^−/−^ 6-OHDA-lesion ∗∗*p* = 0.004; *Ppp1r1b*^f/f^-*Drd1a*-*Cre*^−/−^ sham-lesion vs. *Ppp1r1b*^f/f^-*Drd1a*-*Cre*^+/−^ mice 6-OHDA-lesion ∗*p* = 0.015; *Ppp1r1b*^f/f^-*Drd1a*-*Cre*^+/−^ mice sham-lesion vs. *Ppp1r1b*^f/f^-*Drd1a*-*Cre*^+/−^ mice 6-OHDA-lesion ∗∗*p* = 0.005) and the inactive (C) phase. Data are represented as mean ± SEM. (D and E) Upper panels show hypnograms illustrating the distribution of NREM episodes in *Ppp1r1b*^f/f^-*Drd1a*-*Cre*^−/−^ sham-lesion, *Ppp1r1b*^f/f^-*Drd1a*-*Cre*^−/−^ 6-OHDA-lesion, *Ppp1r1b*^f/f^-*Drd1a*-*Cre*^+/−^ sham-lesion, and *Ppp1r1b*^f/f^-*Drd1a*-*Cre*^+/−^ mice 6-OHDA-lesion mice across the active (D) and inactive (E) phases. Bottom panels show the number and length of NREM sleep bouts in *Ppp1r1b*^f/f^-*Drd1a*-*Cre*^−/−^ sham-lesion, *Ppp1r1b*^f/f^-*Drd1a*-*Cre*^−/−^ 6-OHDA-lesion, *Ppp1r1b*^f/f^-*Drd1a*-*Cre*^+/−^ mice sham-lesion and *Ppp1r1b*^f/f^-*Drd1a*-*Cre*^+/−^ mice 6-OHDA-lesion mice during the active (D, *number of bouts*: the two-way ANOVA showed a significant effect of treatment F (1, 32) = 50.69; ∗∗∗*p* < 0.001, genotype ns, interaction ns; Multiple comparison: *Ppp1r1b*^f/f^ sham-lesion vs. *Ppp1r1b*^f/f^ 6-OHDA-lesion ∗∗∗*p* < 0.001; *Ppp1r1b*^f/f^-*Drd1a*-*Cre*^−/−^ sham-lesion vs. *Ppp1r1b*^f/f^-*Drd1a*-*Cre*^+/−^ mice 6-OHDA-lesion ∗∗*p* = 0.001; *Ppp1r1b*^f/f^-*Drd1a*-*Cre*^+/−^ mice sham-lesion vs. *Ppp1r1b*^f/f^-*Drd1a*-*Cre*^+/−^ mice 6-OHDA-lesion ∗∗∗*p* < 0.001; *mean length*: the two-way ANOVA showed a significant effect of treatment F (1, 32) = 18.64; ∗∗∗*p* < 0.001, genotype ns, interaction ns; Multiple comparison: *Ppp1r1b*^f/f^-*Drd1a*-*Cre*^−/−^ sham-lesion vs. *Ppp1r1b*^f/f^-*Drd1a*-*Cre*^−/−^ 6-OHDA-lesion ∗∗*p* = 0.005; *Ppp1r1b*^f/f^-*Drd1a*-*Cre*^−/−^ sham-lesion vs. *Ppp1r1b*^f/f^-*Drd1a*-*Cre*^+/−^ mice 6-OHDA-lesion ∗*p* = 0.046) and the inactive (E, *number of bouts*: the two-way ANOVA showed a significant effect of treatment F (1, 32) = 76.12; ∗∗∗*p* < 0.001, genotype F (1, 32) = 9.697; ∗∗*p* = 0.004, interaction ns; Multiple comparison: *Ppp1r1b*^f/f^-*Drd1a*-*Cre*^−/−^ sham-lesion vs. *Ppp1r1b*^f/f^-*Drd1a*-*Cre*^+/−^ mice sham-lesion ∗*p* = 0.030; *Ppp1r1b*^f/f^-*Drd1a*-*Cre*^−/−^ sham-lesion vs. *Ppp1r1b*^f/f^-*Drd1a*-*Cre*^−/−^ 6-OHDA-lesion ∗∗∗*p* < 0.001; *Ppp1r1b*^f/f^-*Drd1a*-*Cre*^−/−^ sham-lesion vs. *Ppp1r1b*^f/f^-*Drd1a*-*Cre*^+/−^ mice 6-OHDA-lesion ∗∗*p* = 0.002 *Ppp1r1b*^f/f^-*Drd1a*-*Cre*^+/−^ mice sham-lesion vs. *Ppp1r1b*^f/f^-*Drd1a*-*Cre*^+/−^ mice 6-OHDA-lesion ∗∗∗*p* < 0.001; *mean length*: the two-way ANOVA showed a significant effect of treatment F (1, 32) = 70.22; ∗∗∗*p* < 0.001, genotype F (1, 32) = 10.62; ∗∗*p* = 0.003, interaction ns; Multiple comparison: *Ppp1r1b*^f/f^-*Drd1a*-*Cre*^−/−^ sham-lesion vs. *Ppp1r1b*^f/f^-*Drd1a*-*Cre*^+/−^ mice sham-lesion ∗∗*p* = 0.006; *Ppp1r1b*^f/f^-*Drd1a*-*Cre*^−/−^ sham-lesion vs. *Ppp1r1b*^f/f^-*Drd1a*-*Cre*^−/−^ 6-OHDA-lesion ∗∗∗*p* < 0.001; *Ppp1r1b*^f/f^-*Drd1a*-*Cre*^−/−^ sham-lesion vs. *Ppp1r1b*^f/f^-*Drd1a*-*Cre*^+/−^ mice 6-OHDA-lesion ∗∗*p* = 0.006; *Ppp1r1b*^f/f^-*Drd1a*-*Cre*^+/−^ mice sham-lesion vs. *Ppp1r1b*^f/f^-*Drd1a*-*Cre*^+/−^ mice 6-OHDA-lesion ∗∗∗*p* < 0.001) phase. Bonferroni post-hoc test. Data are represented as mean ± SEM.

In a parallel study, wakefulness and sleep states were assessed in D1-D32^+^ and DARPP-32-deficient D1-D32^-^ mice, following sham- or 6-OHDA-lesion. Consistent with the observations in A2A-D32^+^ mice, 6-OHDA lesioning of D1-D32^+^ mice led to an increase in NREM sleep during the active phase (Figures 6A and 6B). This effect was driven by a marked enhancement in the number of NREM sleep bouts (Figure 6D, bottom left panel), accompanied by a reduction in mean bout duration (Figure 6D, bottom right panel). Similar patterns were observed in 6-OHDA-lesion D1-D32^-^ mice when compared with both sham-lesion D1-D32^+^ and D1-D32^-^ mice (Figures 6A and 6B).

During the inactive phase, 6-OHDA lesioning of D1-D32^+^ mice did not alter total NREM sleep time (Figure 6C) but led to an increase in the number of NREM sleep bouts, accompanied by a significant reduction in their mean duration (Figure 6E). These opposing changes resulted in a total NREM sleep amount comparable to that of sham-lesion D1-D32^+^ controls (Figure 6C). Despite the reduced number and increased duration of NREM sleep bouts caused by DARPP-32 deletion in D1R-expressing neurons (Figure 4E), 6-OHDA injection in D1-D32^-^ mice still induced sleep fragmentation, as evidenced by the increased number and shorter duration of NREM bouts compared with control sham lesion D1-D32^+^ and D1-D32^-^ mice (Figure 6E).

### Istradefylline counteracts excessive daytime sleepiness by reducing the number of non-rapid eye movement sleep episodes

The results obtained in transgenic mice indicate that the loss of DARPP-32 signaling in A2AR-expressing neurons confers protection against PD-related sleep disturbances. Given the positive regulation of DARPP-32 by A2AR, we hypothesized that the pharmacological inhibition of these receptors might similarly counteract excessive daytime sleepiness, as observed in 6-OHDA-lesioned A2A-D32− mice (Figures 5A–5C). To explore this possibility, we evaluated, in the mouse model of PD, the effect of the A2AR antagonist, Istradefylline.^28^^,^^29^

**Figure 7:**
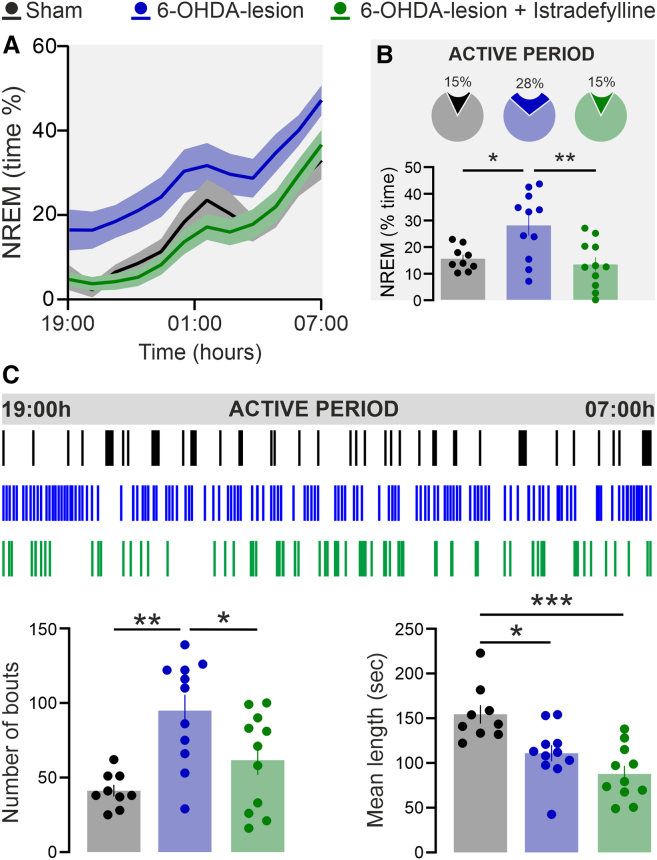
*Istradefylline counteracts EDS by reducing the number of NREM sleep episodes* (A) Time course of NREM sleep in sham-lesion (Sham), 6-OHDA-lesion, and 6-OHDA-lesion + Istradefylline mice, during the 12-h active phase of the circadian cycle (graph is expressed as % over total recording time). (B) Pie chart (top) and bar graph (bottom) show the average NREM sleep time spent by Sham (*n* = 9), 6-OHDA-lesion (*n* = 11), and 6-OHDA-lesion + Istradefylline (*n* = 11) mice during the active phase (one-way ANOVA showed a significant effect *p* = 0.002, F_2,28_ = 7.459; ∗*p* = 0.020 6-OHDA-lesion vs. Sham, ∗∗*p* = 0.004 6-OHDA-lesion + Istradefylline vs. 6-OHDA-lesion). Data are represented as mean ± SEM. (C) Upper panel shows hypnograms illustrating the distribution of NREM episodes in Sham, 6-OHDA-lesion, and 6-OHDA-lesion + Istradefylline mice during the 12-h active phase. Bottom panels show the number and length of NREM sleep bouts in Sham, 6-OHDA-lesion, and 6-OHDA-lesion + Istradefylline mice during the 12-h active (*number of bouts*: the one-way ANOVA showed a significant effect *p* = 0.001, F_2,28_ = 8.670, ∗∗*p* = 0.001 6-OHDA-lesion vs. Sham, ∗*p* = 0.038 6-OHDA-lesion + Istradefylline vs. 6-OHDA-lesion; *mean length*: the one-way ANOVA showed a significant effect *p* < 0.001, F_2,28_ = 12.07; ∗*p* = 0.011 6-OHDA-lesion vs. Sham, ∗∗∗*p* < 0.001 6-OHDA-lesion + istradefylline vs. Sham). Bonferroni post-hoc test. Data are represented as mean ± SEM.

Because DARPP-32 deletion in A2AR-expressing neurons specifically abolished 6-OHDA-induced excessive daytime sleepiness, sleep analyses were focused on the 12-h active phase. As expected, 6-OHDA lesions increased the time spent in NREM sleep in naive mice (Figures 7A and 7B) and produced a marked rise in the number of NREM sleep bouts, accompanied by a reduction in their mean duration (Figure 7C).

Notably, the systemic administration of the A2AR antagonist Istradefylline restored normal NREM sleep duration in 6-OHDA-lesion mice (Figures 7A and 7B). This effect was achieved by counteracting the increase in NREM bout number, while the reduction in bout length persisted (Figure 7C, bottom panels). Thus, the pharmacological inhibition of A2AR closely recapitulated the effects of genetic DARPP-32 deletion in A2AR-expressing neurons.

## Discussion

In this study, we identify DARPP-32, a dopamine signaling protein highly expressed in striatal neurons, as a critical regulator of sleep-wake function and a potential therapeutic target for PD-related sleep impairment. The deletion of DARPP-32 in two distinct populations of SPN produced divergent quantitative and qualitative changes affecting NREM sleep. Specifically, loss of DARPP-32 in A2AR-expressing SPN reduced NREM sleep during the active phase of the circadian cycle, whereas DARPP-32 deficiency in D1R-expressing SPN led to the excessive stabilization of NREM sleep during the inactive phase. Importantly, in a mouse model of PD, the targeted ablation of DARPP-32 in A2AR-expressing SPN mitigated sleep dysfunction by abolishing EDS.

Previous work demonstrated that the cell type-specific deletion of DARPP-32 using a D2R-Cre driver enhances locomotor activity in a novel environment.^21^ Consistent with the selective co-expression of A2AR and D2R in SPN,^30^ we observed a similar effect when DARPP-32 deletion was driven by an A2AR-Cre line.^31^^,^^32^ However, following habituation, the time spent in locomotion by A2A-D32^-^ mice returned to the control level. Therefore, the increase in wakefulness observed in these mice cannot be attributed to hyperlocomotion but rather reflects a reduction in NREM sleep, likely resulting from impaired DARPP-32-PKA signaling and consequent inhibition of A2AR-expressing SPNs.

In mice, alterations in the activity of SPN co-expressing A2AR and D2R have been shown to modulate sleep-wake states. Chemogenetic inhibition of these neurons in the dorsal striatum, which is largely innervated by dopaminergic neurons from the SNc, reduces NREM sleep and thereby increases wakefulness during the active phase.^13^ A comparable effect has been observed following the activation of midbrain dopamine neurons located in the VTA. In this case, the increase in wakefulness results from the activation of inhibitory D2R located in the SPN of the nucleus accumbens.^33^ The present study suggests that these manipulations converge on a common intracellular mechanism, promoting wakefulness and reducing NREM sleep through the suppression of DARPP-32 signaling, which is abundantly expressed in both dorsal and ventral SPN.

Consistent with previous work demonstrating opposite roles of striatal D1R- and D2R-expressing neurons on motor control,^21^ ablation of DARPP-32 in D1R-expressing SPN reduced locomotor activity in the open field. This reduction was confined to the active phase of the circadian cycle and persisted after habituation to the novel environment. Despite the altered locomotor behavior, DARPP-32-deficient mice showed no change in total wake-sleep duration across the 24-h recording period. This observation contrasts with recent work showing reduced wakefulness following the chemogenetic inhibition of D1R-expressing striatal neurons.^9^ The discrepancy may reflect fundamental differences between the rapid, temporally restricted effect of chemogenetic manipulation and the long-term alteration of dopamine signaling induced by constitutive loss of DARPP-32.

Although D1-D32^−^ and D1-D32^+^ mice exhibited comparable total NREM sleep duration, they differed markedly in sleep architecture. Polysomnographic recording revealed that DARPP-32-deficient mice displayed fewer NREM sleep episodes of longer duration, compared to D1-D32^+^ control littermates. Notably, this difference was restricted to the inactive phase of the circadian cycle, indicating enhanced consolidation and stabilization of nocturnal sleep in the absence of DARPP-32.

The alterations in wake-sleep duration and architecture caused by the selective ablation of DARPP-32 in A2AR- or D1R-expressing SPN prompted us to investigate whether these interventions influence PD-related sleep dysfunctions. To address this question, we employed a mouse model that reproduces EDS and sleep fragmentation, two of the most prominent sleep disturbances associated with PD.^16^ Using this model, we found that the suppression of DARPP-32 in striatal neurons co-expressing A2AR and D2R abolished EDS. Given the central role of DARPP-32 in A2AR-mediated signaling,^25^ this effect is most likely mediated by normalizing excessive A2AR activity generated, in PD, by the loss of D2R antagonistic control.^6^ To substantiate this hypothesis, we examined the effects of istradefylline, an A2AR antagonist known to reduce cAMP-dependent DARPP-32 signaling,^34^ in the PD model. The systemic administration of istradefylline reduced daytime sleepiness to a degree comparable to that observed following DARPP-32 ablation in A2AR-expressing SPNs. Overall, these findings support the idea that DARPP-32 and related downstream signaling effectors may constitute promising therapeutic targets for the treatment of sleep comorbidities in PD.

Our results indicating that EDS can be controlled by counteracting A2AR overactivation in SPN are partially consistent with the observation that, in the same PD model, the systemic administration of the D2R agonist, pramipexole, reduces daytime sleepiness.^16^ However, this protective effect is transient and is followed, during the late phase of the active period, by a prolonged rebound of intensified NREM sleep, ultimately exacerbating EDS.^16^ Taken together, these observations suggest that a sustained and stable reduction of A2AR-mediated signaling, such as that achieved through the suppression of its downstream effector DARPP-32, provides a more strategy for managing of PD-related EDS.

The results of the studies in the PD model indicate that the protective effect on sleep dysfunction occurs only in combination with the loss of DARPP-32 in a subpopulation of SPN co-expressing A2AR and D2R. Accordingly, while the depletion of DARPP-32 in D1R-expressing neurons in naive mice increases the duration of sleep episodes during the inactive phase, the same manipulation fails to normalize sleep fragmentation in the PD model. This dissociation suggests that, in Parkinsonian conditions, the disruption of NREM sleep stability may occur independently of impaired dopaminergic transmission. In this context, it is important to note that the PD model used in this study is based on 6-OHDA, which targets both dopaminergic and noradrenergic neurons.^35^ Therefore, any potential protective effect on sleep fragmentation mediated by DARPP-32 deletion in D1R-expressing SPNs may be offset by the concomitant disruption of noradrenergic signaling.

In conclusion, this study demonstrates that DARPP-32, a central mediator of PKA-dependent transmission highly expressed in distinct populations of dopaminoceptive SPN, plays a critical role in regulating fundamental features of sleep architecture by controlling the duration and stability of NREM sleep. Importantly, our findings further show that EDS, a major sleep disorder associated with PD, can be corrected by silencing DARPP-32 in a specific population of SPN co-expressing A2AR and D2R. Together, these results provide a rationale for the development of novel approaches aimed at managing PD-related sleep comorbidities through the cell type-specific modulation of intracellular targets within the PKA-DARPP-32 signaling cascade.

### Limitations of the study

wThe *Drd1a*-Cre and *Adora2a*-Cre driver lines used in this study may mediate Cre recombinase expression in D1R- and A2AR-expressing neurons located outside the striatum. Although this limitation is partially mitigated by the predominant expression of DARPP-32 in SPNs and by the well-established involvement of nucleus accumbens A2AR signaling in sleep-wake regulation,^14^^,^^15^ potential off-target effects cannot be entirely excluded. In this regard, the conclusions of the present study would be further strengthened by future experiments aimed at selectively rescuing DARPP-32 expression in D1R- and A2AR-expressing SPNs.

The mouse model of PD employed in this study did not exhibit alterations in REM sleep duration or architecture.^16^ Consequently, the impact of DARPP-32 ablation in D1R- and A2AR-expressing neurons on sleep disturbances was primarily evaluated through the analysis of NREM sleep. Additional studies will be required to determine whether DARPP-32 also contributes to the regulation of REM sleep. Furthermore, future investigations will be necessary to delineate the specific DARPP-32–dependent signaling pathways that underlie the attenuation of EDS in Parkinson’s disease.

## Resource availability

### Lead contact

Requests for further information and resources should be directed to and will be fulfilled by the lead contact, Gilberto Fisone (gilberto.fisone@ki.se).

### Materials availability

This study did not generate new unique reagents.

### Data and code availability

The original code utilized in the study has been deposited at GitHub at https://doi.org/10.1016/j.pneurobio.2023.

Any additional information required to reanalyze the data reported in this article is available from the lead contact upon request.

## Acknowledgments

This work was supported by the 10.13039/501100004359Swedish Research Council (VR-2024-02966) and the Swedish Parkinson Foundation (nr. 1561/24). The behavioral studies were conducted with support from the Animal Behavioral Core Facility (ABCF) at 10.13039/501100004047Karolinska Institutet.

## Author contributions

Conceptualization, C.A.P. and G.F.; methodology, C.A.P.; investigation, C.A.P., M.L.S., and A.R.; writing – original draft, G.F. and C.A.P.; writing – review and editing, G.F. and C.A.P.; funding acquisition, G.F.; resources, G.F.; supervision, G.F.

## Declaration of interests

The authors declare no conflict of interest.

## STAR★Methods

### Key resources table

**Table:** 

REAGENT or RESOURCE	SOURCE	IDENTIFIER
**Antibodies**		

Alexa Fluor Guinea Pig 488	Jackson Immuno Research	106-545-003
Alexa Fluor Mouse 594	Jackson Immuno Research	115-585-146
Alexa Fluor Rabbit 594	Jackson Immuno Research	111-585-003
Guinea Pig Anti-Cre recombinase	SYSY	257–005
Mouse Anti-β-actin	Sigma-Aldrich	A5441
Mouse Anti-DARPP-32	Gifted by P.Greengard, Rockefeller University	–
Rabbit Anti-Tyrosine Hydroxilase	Millipore	AB152

**Chemicals, peptides, and recombinant proteins**		

6-OHDA HCl	Sigma-Aldrich	H4381
Istradefylline	Tocris	5147

**Software and algorithms**		

MAT Lab Code Sleep Analysis	https://github.com/dacamemg/Sleep_Sorting_Karolinska.git	https://doi.org/10.1016/j.pneurobio.2023

### Experimental model details

Mice of both sexes (10–12 weeks) with conditional deletion of the gene coding for DARPP-32 (*Ppp1r1b*) in D1R- or A2AR-expressing neurons were generated by breeding *Ppp1r1b* floxed mice with *Drd1a*-*Cre*^+/−^ mice (*Ppp1r1b*^f/f^-*Drd1a*-*Cre*^+/−^ mice)^21^ or *Adora2a*-*Cre*^+/−^ mice (*Ppp1r1b*^f/f^-*Adora2a*-*Cre*^+/−^ mice) - hereafter referred to as D1-D32^-^ and A2A-D32^-^ mice. *Ppp1r1b*^f/f^-*Drd1a*-*Cre*^−/−^ and *Ppp1r1b*^f/f^-*Adora2a*-*Cre*^−/−^ mice - hereafter referred to as D1-D32^+^ and A2A-D32^+^ mice - were used as control. The driver lines efficacies were confirmed by determining the number of Cre-positive cells expressing DARPP-32 (Figures S1A and S1D).

Mixed sex C57BL/6J mice (10–12 weeks, 20–25 g; Charles River, Germany) were used in the experiments with Istradefylline (Tocris, Bio-Techne, Ireland). Mice were group-housed under 12:12 h light-dark cycle in a climate-controlled environment (22°C) with food and water *ad libitum*. The experiments were conducted in accordance with the guidelines of the Research Ethics Committee of Karolinska Institutet, Swedish Animal Welfare Agency (Ethical Permit 14673-22) and European Community Council Directive 86/609/EEC.

### Method details

#### Drugs

6-Hydroxydopamine hydrochloride (6-OHDA; Sigma-Aldrich, Stockholm, Sweden) was dissolved in 0.9% saline containing 0.02% ascorbic acid, to a free base concentration of 4 μg/μL and injected into the right medial forebrain bundle (MFB).^16^ Istradefylline (Tocris, Bio-techne, Dublin, Ireland) was prepared in 0.9% saline containing 8% Tween-80 and administered intraperitoneally at a dose of 3 mg/kg in a volume of 10 mL/kg, 15 min prior to the onset of the active phase.^34^^,^^36^

#### Analysis of motor activity

Horizontal motor activity was examined in the open field test previously described^37^ using a square area (45 × 45 × 39 cm) with light gray floor and clear Plexiglass walls surrounded by two frames of light beams placed at 3 cm above the floor of the arena to detect movements (ActiMot system; TSE Systems, Germany). Explorative behavior was recorded for 60 min with light level in the center of the floor adjusted to 150–200 lux.

To determine motor activity following habituation, mice were first anesthetized with 4% isoflurane for 60–120 s and a radio-frequency identification (RFID) tag (12 mm in length, 2–3 mm in diameter; Planet-ID GmbH, Germany) was inserted between the scapulae for unique identification. One week later, mice were group-housed in home cages placed on a sensor plate consisting of a 2 × 4 grid of RFID sensors (Activity Monitor, PhenoSys, Berlin, Germany). The 48-h recording began after a 6-h habituation period, to minimize potential confounding effects of novel cage-induced exploratory behavior. Two-dimensional position and movement were tracked for each mouse by identifying its unique RFID tag with a sampling rate of 3 Hz. The X- and Y-coordinates were continuously recorded and stored in CSV format on a PC’s hard drive. Raw data were extracted in hourly bins for each mouse and analyzed as the mean activity index per mouse per hour.^27^

#### *6-*OHDA injection and electrode implant

Mice were anesthetized with isoflurane and positioned in a stereotaxic frame (Stoelting Europe, Dublin, Ireland) provided with a heating pad (37°C) to preserve normothermia. Before surgery, animals received a subcutaneous injection of analgesic (0.1 mg/kg - Temgesic, Apoteket, Sweden) and ophthalmic ointment (Oftagel; Santen, Apoteket, Sweden) to prevent corneal dryness. The lesion was induced by injecting 4 μg of 6-OHDA dissolved in 1 μL of vehicle, in the MFB of the right hemisphere, according to the following coordinates (mm from bregma): anteroposterior (AP), −1.2; mediolateral (ML), −1.2; dorsoventral (DV) − 4.8. Sham-lesion (control) mice were injected with an equivalent volume of vehicle. Two weeks after 6-OHDA injection, the mice were implanted with surface micro-screws positioned over the left parietal cortex (AP: −2.0 and ML: 1.8 mm from bregma - contralateral to the side of 6-OHDA injection) and in the occipital bone for cortical activity recording and signal reference. Stainless-steel teflon-coated electrodes (Model 791400; A-M Systems Inc., Carlsborg, WA, USA) were implanted over the neck muscles for electromyographic (EMG) recording. The electrode and micro-screws were then soldered to a connector (Straight Male PCB Header - 4) fixed to the skull with dental cement and acrylic, and mice were allowed to recover for one week.^16^

#### Polysomnographic and behavioral recording

Cortical and muscle electrical activity were amplified (5000 × gain) and bandpass filtered at 1 Hz high pass for all channels, low pass filters set as 3 kHz for electroencephalography (EEG) and 500 Hz for electromyography (EMG) by signal conditioners (ERS100C and EMG 100 C amplifiers - Biopac MP160). The signals were recorded at a sample rate of 6250 Hz using AcqKnowledge software (version 4.1, Biopac Systems Inc, CA, USA) and stored for offline analyses. Genetically modified, DARPP-32 deficient, mice underwent two consecutive recording sessions, encompassing a 3-day total recording period. Mice utilized for the pharmacological experiment with Istradefylline, underwent an additional day of recording, following drug injection. On day one, the animals were habituated to the room and the recording system from 10:00 to 16:00 h. Recording started the following day at 18:00 h and lasted until the next day at 19:00 h. The full 24-h cycle recording consisted of 12 h lights-off (from 19 to 7 h, corresponding to the active period) followed by 12 h lights-on (from 7 to 19 h, corresponding to the inactive period) discarding the first hour to avoid distress interference. Each recording session was performed in freely moving mice and placed into adjacent separate cages provided with bedding, nest material, food, and water. Mice behavior during polysomnography was recorded by Logitech Brio webcams at 1080 pixels. EEG, EMG, and behavioral recordings were combined in a single video file using OBS Studio software (Version 27.0.1) at ten frames per second.^16^

#### State sorting

The combination of polysomnographic and behavioral recording allowed for precise state sorting and sleep architecture analysis.^16^ The 24-h period was scored manually into 10-s epochs of awake, NREM, or REM state. Awake state was defined by desynchronized low amplitude EEG and by sustained EMG activity encompassing movement and stillness periods. NREM sleep was identified based on a relative increase in EEG amplitude mainly consisting of slow waves, along with low EMG tone, and lack of movement. REM state was distinguished by immobility, stable theta-based EEG patterns, and absence of EMG activity.

#### Analysis of sleep architecture

The analysis of sleep architecture and cortical activity was performed by a custom script developed in MATLAB^16^ (code available at: https://github.com/dacamemg/Sleep_Sorting_Karolinska.git). The distribution and time spent in awake, NREM, and REM states across the 24-h cycle was calculated in 1-h bins and averaged for 6 or 12 h periods, according to the light off/on time-point (19–7 h and 7–19 h, respectively). NREM sleep fragmentation was assessed in 1-h bins by measuring the number and length of bouts and averaged for 12-h periods. In this study, only the data from NREM were presented.

#### Perfusion and immunofluorescence analysis

After recording, the mice were deeply anesthetized with 60 mg/kg pentobarbital (Sanofi-Aventis, France; diluted 1:1 in 0.9% saline solution; i.p.) and transcardially perfused with ice-cold phosphate-buffered saline (PBS) followed by 4% (weight/vol) paraformaldehyde (PFA; Sigma Aldrich, Stockholm, Sweden) in 0.1 M phosphate buffer (pH 7.4). Brains were then post-fixed overnight in 4% PFA at 4°C, rinsed in PBS and cut into 40 μm sections with a vibratome (VT1000S, Leica, Germany). Sections from striatum and midbrain were collected and stored at −20°C in cryoprotectant solution (25% glycerol, 30% ethylene glycol, 0.1 M PBS, pH 7.2) until analysis. The 6-OHDA lesion was confirmed by immunofluorescence against tyrosine hydroxylase (TH) (Figure S2). Sections were treated with PBS + Triton 0.3% and incubated in a blocking solution of normal serum 10% and bovine serum albumin 1% for 1 h at room temperature. Sections were then incubated overnight at 4°C with a mouse DARPP-32 antibody (1:10000, Gifted by P.Greengard, Rockefeller University), a rabbit TH antibody (1:1000, Millipore, Germany) or a Guinea Pig Cre recombinase antibody (1:500, SYSY, Germany). The following day, the sections were washed and incubated with the appropriate secondary antibody (Alexa Fluor; Thermo Fisher Scientific, Sweden). The sections were imaged using a confocal microscope (LSM 800; ZEISS, Germany) at 20X or 10X. Only animals showing a 90% or higher depletion of TH were used for the PD study (Figure S2B).

#### Western blot

Striata were rapidly dissected, sonicated in 1% sodium dodecyl sulfate, and boiled for 10 min. Equal amounts of protein (25 μg) from each sample were loaded onto 10% polyacrylamide gels, separated by electrophoresis, and transferred overnight to nitrocellulose membranes (Thermo Fisher Scientific, Sweden). The membranes were immunoblotted with primary antibodies against actin (1:30000, Sigma Aldrich, Sweden), DARPP-32 (1:10000, Gifted by P.Greengard, Rockefeller University) or TH (1:2000, Millipore, Germany). Detection was based on fluorescent secondary antibody binding (IR Dye 800CW and 680RD; Li-Cor, NE, USA) and quantified using a Li-Cor Odyssey infrared fluorescent detection system (Li-Cor, NE, USA). DARPP-32 or TH protein levels were normalized by the amount of the corresponding actin detected in the sample and then expressed as a percentage of control. Only animals showing a 90% or higher depletion of TH were used (Figure S2C).

### Quantification and statistical analysis

All data were analyzed using GraphPad Prism 9 (GraphPad Software). Depending on the experimental design, comparisons were performed using unpaired *t* test and two-Way ANOVA, as appropriate. When ANOVA yielded significant results, Bonferroni post-hoc tests were applied for multiple comparisons. Statistical significance was defined as *p* < 0.05.
