# Supplementary material for: Identification of DARPP-32 as a novel sleep regulator in physiological conditions and experimental Parkinsonism
Source: iScience. 2026 Feb 2;29(3):114882. doi: 10.1016/j.isci.2026.114882 (PMC12925226; doi:10.1016/j.isci.2026.114882)
Supplement: Document S1. Figures S1 and S2 [file mmc1.pdf]

iScience, Volume *29*

Supplemental information

Identification of DARPP-32 as a novel sleep regulator in physiological conditions and experimental Parkinsonism

Clarissa Anna Pisanò, Maria Laura Santino, Alice Russotto, and Gilberto Fisone

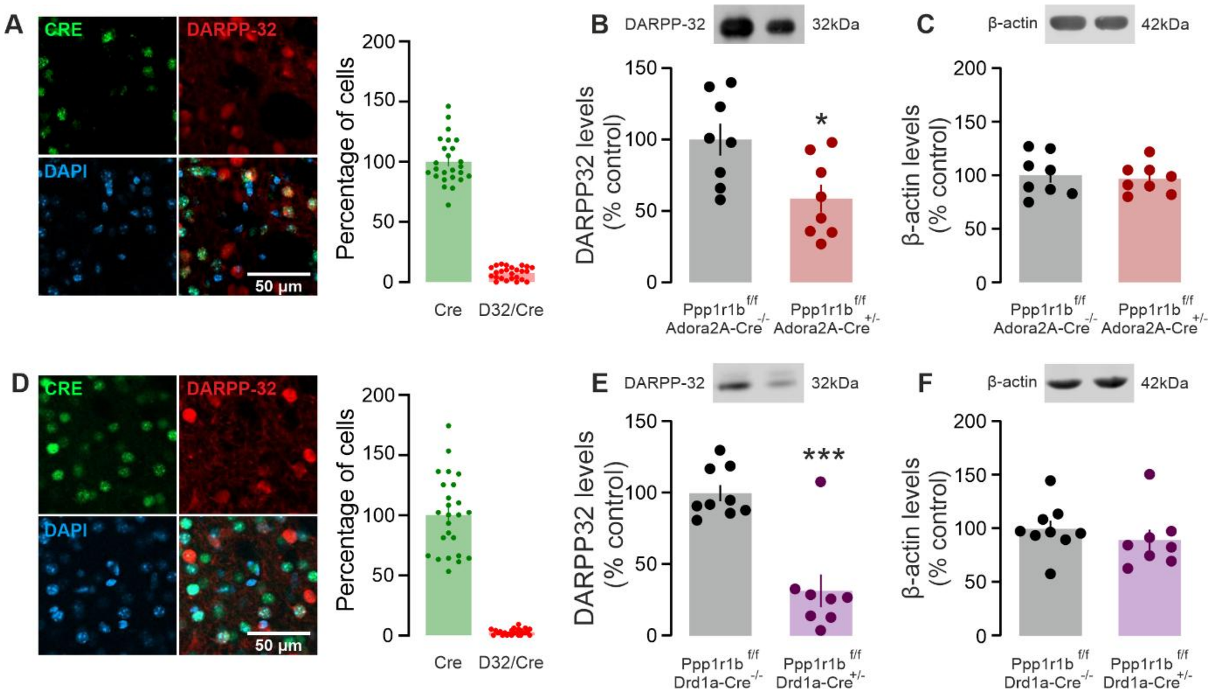

Figure S1. Quantification of Cre–DARPP-32 colocalization and DARPP-32 immunoreactivity in the striatum.

(A, D) Representative immunofluorescence of striatal sections from Ppp1r1bf/f-Adora2A-Cre-/-, Ppp1r1bf/f-Adora2A-Cre+/- mice (A) and Ppp1r1bf/f-Drd1a-Cre-/- and Ppp1r1bf/f-Drd1a-Cre+/- mice stained with antibodies against Cre (green), DARPP-32 (red), and DAPI (blue). Lower right images were obtained merging Cre and DARPP-32 (Scale bar 50 µm). Right panels are the quantification of the percentage of Cre-positive cells expressing DARPP-32 in Ppp1r1bf/f-Adora2A-Cre+/- (A; n=25 images from 4 mice; 8%±0.01%) and Ppp1r1bf/f-Drd1a-Cre+/- (D; n=25 images from 5 mice; 3%±0.004%). (B, C, E, F) Upper panels: representative Western blots of DARPP-32 and β-actin in Ppp1r1bf/f-Adora2A-Cre-/- and Ppp1r1bf/f-Adora2A-Cre+/- mice (B, C) and in Ppp1r1bf/f-Drd1a-Cre-/- and Ppp1r1bf/f-Drd1a-Cre-Cre+/- mice (E, F). Lower panels: bar graphs showing the levels of DARPP-32 and β-actin (B: *p=0.0148 vs. Control; Unpaired t-test, t=2.777, df=14, n=8/group; E: ***p<0.001 vs. Control; Unpaired t-test, t=5.504, df=15; n=8-9/group) and β-actin (B, D). The data are calculated as percent of Control levels and expressed as mean ± SEM.

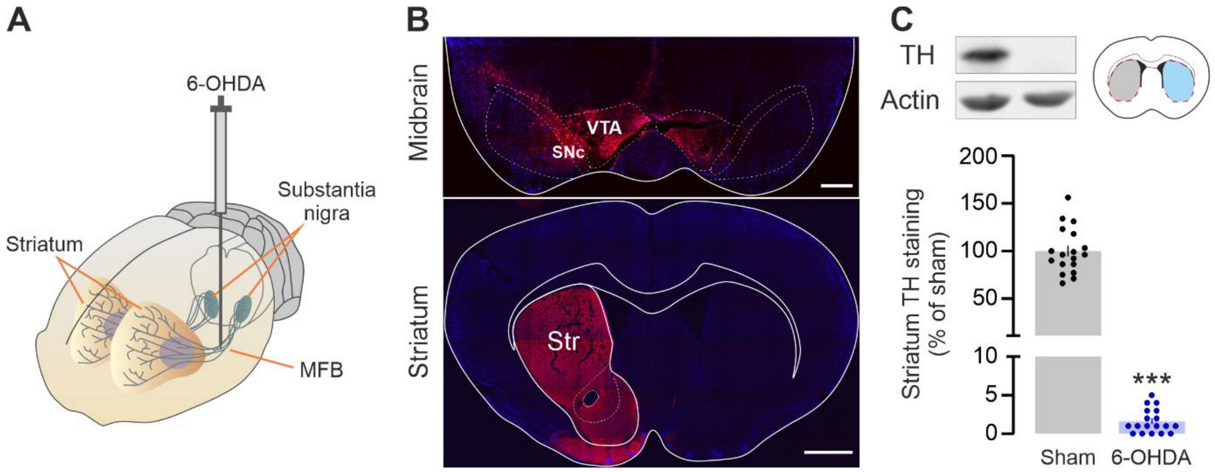

Figure S2. 6-OHDA lesion of MFB.

(A) Schematic representation of the 6-OHDA injection site in the MFB. (B) Representative confocal images from a 6-OHDA injected mouse, showing loss of TH-immunoreactive neurons in the substantia nigra pars compacta (SNc) and ventral tegmental area (VTA) (upper panel, Scale bar 100 μm), as well as degeneration of TH-positive efferent projections in the dorsal striatum (Str) (lower panel, Scale bar 1 mm). (C) Quantification of TH-immunoreactivity in the striatum of sham-lesion and 6-OHDA-lesion mice. Upper panel: representative Western blot showing TH and actin immunoreactivity. Lower panel: bar graph showing data calculated as percent of sham and expressed as mean ± SEM. ***$p<0.001$ vs. Sham; Unpaired T-test, $t=17.29$, $df=34$; $n=18$/group.
